# Ibrutinib-Associated Cardiac Tamponade with Concurrent Antiplatelet Therapy

**DOI:** 10.1155/2020/4282486

**Published:** 2020-03-27

**Authors:** Jennifer L. Miatech, John H. Hughes, Dillon K. McCarty, M. Patrick Stagg

**Affiliations:** Baton Rouge General Internal Medicine Residency Program, Baton Rouge General Medical Center, Baton Rouge, LA, USA

## Abstract

Ibrutinib is approved for the first-line treatment of chronic lymphocytic leukemia (CLL). A well-known side effect of ibrutinib therapy is increased bleeding risk, which ranges from mild mucocutaneous bleeding to rarely life-threatening hemorrhage. The increased bleeding tendency associated with ibrutinib is thought to be related to its effect on several platelet signaling pathways, which can be exacerbated in the setting of concurrent antiplatelet or anticoagulant therapy. We present an 82-year-old male with CLL on ibrutinib and concurrent antiplatelet therapy who developed cardiac tamponade due to a hemorrhagic pericardial effusion requiring emergent placement of a pericardial window. This case further highlights the risk of major bleeding in patients treated with ibrutinib and concurrent antiplatelet therapy.

## 1. Introduction

Chronic lymphocytic leukemia (CLL) is a mature lymphocytic neoplasm resulting in progressive accumulation of monoclonal lymphocytes. CLL is an extremely heterogeneous disease that in a majority of patients is asymptomatic. Patients may present with painless lymphadenopathy or constitutional symptoms such as fevers, night sweats, and weight loss. The most common complications associated with CLL include anemia, thrombocytopenia, and infection.

Ibrutinib, an irreversible inhibitor of Bruton's tyrosine kinase (Btk), is approved as a first-line treatment of CLL. An established side effect of ibrutinib therapy is bleeding; most often it is mild mucocutaneous bleeding and rarely is life-threatening hemorrhage. The risk of major bleeding is a rare complication, estimated to be less than 1–7% [1, 2]. The increased bleeding tendency is partially thought to be related to ibrutinib's inhibition of several signal transduction pathways involved in platelet function.

The concurrent use of anticoagulant or antiplatelet therapy with ibrutinib is not recommended; however, specific guidelines concerning comprehensive use of these agents have yet to be established. Dual antiplatelet therapy is avoided in most other clinical settings due to the increased risk of bleeding. The risk of major bleeding with ibrutinib and aspirin therapy appears similar and possibly more severe than that of the dual antiplatelet therapy often used in post-coronary artery stenting. We present a case of an 82-year-old male with a history of CLL on treatment with ibrutinib and aspirin therapy who developed a life-threatening hemorrhage resulting in cardiac tamponade.

## 2. Case Presentation

An 82-year-old Caucasian male with CLL, gastroesophageal reflux disease, anxiety disorder, gout, hypertension, hyperlipidemia, and obstructive sleep apnea presented to our hospital in May 2019 with complaints of retrosternal chest pain, shortness of breath, and syncope. The patient was seen at another hospital 5 days earlier with similar complaints. Initial work up at the outside hospital included negative troponins, an electrocardiogram showing sinus bradycardia with a rate of 39 beats per minute (bpm), and leukocytosis with anemia (Table 1). Computed tomography (CT) of the chest, abdomen, and pelvis at that time showed an increased number of small retroperitoneal lymph nodes and a small pericardial effusion. He underwent a barium swallow study that revealed normal swallowing with tertiary contractions and mid-esophageal spasms. The patient experienced relief of his retrosternal chest pain with antacid treatment. He was diagnosed at the outside hospital with esophageal spasms and discharged on pantoprazole. He developed postdischarge symptoms of chest pain and shortness of breath that were worse while supine and relieved when leaning forward. A syncopal episode prompted him to present to our hospital.

The patient had a history of CLL Rai Stage 0 that was initially diagnosed in 2013 after routine laboratory studies revealed a white blood count (WBC) of 17 × 10^9^/L with 70% lymphocytes. Flow cytometry and bone marrow biopsy at that time were consistent with CLL. Further work up included normal chromosomal studies and fluorescence in-situ hybridization (FISH) studies negative for 17p deletion. He was initially managed conservatively without treatment until 2015 when his WBC increased to 93 × 10^9^/L. He was then started on obinutuzumab and chlorambucil, but due to medication intolerance chlorambucil had to be discontinued. He completed the course of obinutuzumab in 2016. The WBC initially improved after treatment, but then slowly began to increase in 2017 to 17 × 10^9^/L. Repeat chromosomal studies and FISH at that time were positive for a 17p deletion. Following repeat testing, he was started on ibrutinib 420 mg/day in May 2017. The patient's other home medications included aspirin for primary cardiovascular protection, citalopram, allopurinol, amlodipine, enalapril, spironolactone, and atorvastatin. He had no other medication changes over the previous 24 months.

**Figure 1:**
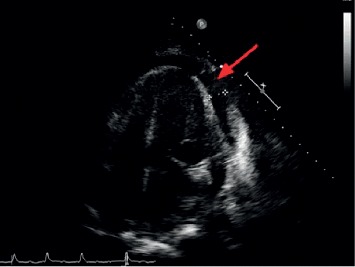
Transthoracic echocardiogram (TTE) demonstrating a large pericardial effusion (red arrow).

On presentation to our hospital, he had a temperature of 97.8°F, heart rate of 132 bpm, respiratory rate of 20 breaths/minute, blood pressure of 98/54 mmHg, and oxygen saturation of 92% on 2 liters of supplemental oxygen. Physical exam was significant for irregular heart rate, muffled heart sounds, and an elevated jugular venous pulse. Laboratory studies revealed a new acute kidney and hepatic injury with an elevated creatinine of 2.43 mg/dL (patient's baseline 1.1 mg/dL), alanine transaminase (ALT) of 93 U/L, aspartate transaminase (AST) of 59 U/L, and alkaline phosphatase of 123 U/L. His CBC (complete blood count) results included a WBC of 16.3 × 10^9^/L with an absolute lymphocyte count of 2.08 × 10^9^/L, hemoglobin of 10.5 g/dL, and a platelet count of 204 × 10^9^/L. Coagulation studies revealed normal findings including a partial thromboplastin time (PTT) of 29 sec, prothrombin time (PT) of 15 sec, and an international normalized ratio (INR) of 1.2. Electrocardiogram showed atrial fibrillation with rapid ventricular response. Chest radiograph revealed an enlarged cardiac silhouette. Emergent echocardiogram revealed a large pericardial effusion measuring 2 cm with associated right ventricular diastolic collapse, consistent with cardiac tamponade (Figure 1).

The patient underwent an emergent pericardial window with removal of 500 mL of bloody pericardial fluid. The pericardial fluid cultures and cytology were negative for infection or malignancy. Repeat echocardiogram performed 3 days after the pericardial window showed a normal left ventricular ejection fraction of 55–65% with normal ventricular chamber size. He was started on treatment for atrial fibrillation with amiodarone, and his ibrutinib and aspirin therapy were discontinued. The patient's acute hepatic and kidney injuries were attributed to hypoperfusion injury in the setting of cardiac tamponade, these studies normalized prior to discharge with a creatinine of 0.9 mg/dL, ALT of 20 U/L, AST of 29 U/L, and alkaline phosphatase of 74 U/L.

Quantitative assessment of platelet function was not performed urgently as both the patient's aspirin and ibrutinib were discontinued given that aspirin use for primary prevention of cardiac disease is not indicated and the patient's CLL was well controlled. The patient remained off antiplatelet and anticoagulant therapy. His WBC remained stable at 7.8 × 10^9^/L with an absolute lymphocyte count of 3.78 × 10^9^/L off ibrutinib therapy 4 months later on outpatient follow-up without further intervention. The patient was in normal sinus rhythm during his 5 month follow-up with cardiology and amiodarone was switched to metoprolol.

## 3. Discussion

CLL is a lymphoproliferative neoplasm that results in the accumulation of mature lymphocytes. CLL is the most common leukemia in the United States, accounting for approximately 25 to 30% of all leukemias [3]. The majority of patients are asymptomatic and are usually diagnosed during routine laboratory testing. Current treatment recommendations for patients with early stage asymptomatic CLL with no poor prognostic features is observation rather than treatment. As the disease progresses, the most common complications include infection, anemia, and thrombocytopenia. Rare potentially life-threatening complications associated with CLL include tumor lysis syndrome, secondary cancers, and major bleeding events. Treatment is recommended in patients who develop disease related complications such as significant constitutional symptoms, bone marrow failure, or other symptomatic manifestations from progressive disease.

In CLL, constitutive activation of tyrosine kinases, including Btk, results in the uncontrolled proliferation and survival of malignant B lymphocytes. Ibrutinib, an irreversible inhibitor of Btk, inhibits B-cell receptor pathways, which prevents further cell growth, proliferation, and survival of malignant B cells [4]. Overall, frontline therapy with ibrutinib is generally well tolerated, with the most common side effects being fatigue, diarrhea, and myalgias. More serious complications include atrial fibrillation, reported in up to 16% of patients on ibrutinib therapy [5]. Our patient's atrial fibrillation was not attributed to ibrutinib as it developed in the setting of pericardial tamponade.

Ibrutinib also has a known association with bleeding, which most commonly manifests as mucocutaneous bleeding from mild petechia to actinic purpura. Although rarely reported, the bleeding can sometimes present as life-threatening hemorrhage, most often in the form of central nervous system hemorrhage [5]. Our patient presented with major hemorrhage in the form of cardiac tamponade while on ibrutinib and aspirin therapy. Hemorrhagic pericardial effusions are most often secondary to malignancy, trauma (including percutaneous interventional procedures), myocardial infarction, and idiopathic causes [6]. Few cases in the literature have described the development of spontaneous hemorrhagic cardiac tamponade in patients with CLL, with the majority of these patients being on anticoagulation [7, 8].

**Figure 2:**
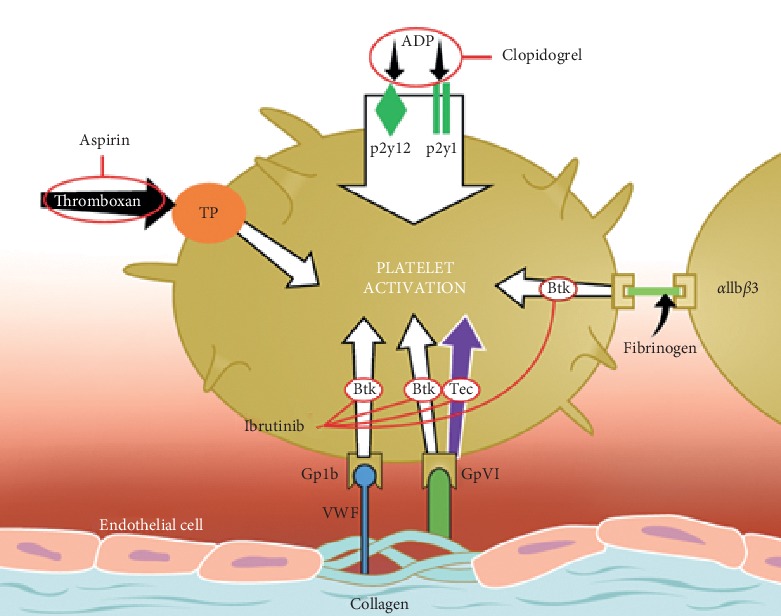
The effects of ibrutinib on platelet function. Btk, Bruton's tyrosine kinase; Tec, tyrosine kinase expressed in hepatocellular carcinoma; aspirin effects thromboxane A2 receptor (TP); clopidogrel effects adenosine diphosphate (ADP) via p2y12 & p2y1 receptors; von Willebrand factor (VWF) effects glycoprotein Ib (GpIb); collagen binds glycoprotein VI (GpVI); fibrinogen binds integrin *α*IIb*β*3. This work, “The effects of ibrutinib on platelet function,” is a derivative of “The effects of ibrutinib on platelets” by Shatzel et al., retrieved from https://onlinelibrary.wiley.com/doi/full/10.1111/jth.13651. Used under Creative Commons Attribution-NonCommercial 3.0 Unported (CC BY-NC 3.0) Generic license https://creativecommons.org/licenses/by-nc/3.0/.

The increased tendency for bleeding as a result of ibrutinib therapy is thought to be secondary to its effect on several antiplatelet mechanisms, notably the inhibition of Btk and Tec (tyrosine kinase expressed in hepatocellular carcinoma) kinases [2]. Btk and Tec kinases both play a major role in platelet activation downstream of the collagen glycoprotein VI (GpVI) and glycoprotein Ib (GpIb) (Figure 2). Current studies suggest that there is an overall increased risk of bleeding, with no significant risk of life-threatening hemorrhage compared to the general population [9]. However, a more recent retrospective study evaluating the risk of major bleeding in patients on ibrutinib found that this may be higher than previously thought, especially in the setting of concurrent antiplatelet or anticoagulant use [10]. Thrombocytopenia and platelet dysfunction as a result of CLL are other factors that may contribute to the varying degrees of bleeding. Many patients treated for CLL are on aspirin therapy for cardiovascular prevention. Reported clinical trial data in patients with mantle cell lymphoma suggest that the addition of antiplatelet agents to ibrutinib resulted in an increased risk of bleeding [2]. Major hemorrhage was demonstrated in early clinical trials when both ibrutinib and vitamin K antagonists were used together. This resulted in the exclusion of patients on warfarin from participating in further trials [1]. Currently, there have been no clinical trials investigating the risk of increased bleeding specifically in patients with CLL on ibrutinib and antiplatelet therapy; though, clinicians are advised to use caution when using these therapeutic agents together [1].

The increased bleeding risk associated with ibrutinib, especially in the setting of concurrent antiplatelet or anticoagulant therapy, has led to the development of several treatment strategies used to reduce the risk. No consensus guidelines on the combination of other anticoagulants and ibrutinib use exist. An approach we have used is to reduce the dose of ibrutinib and anticoagulants by 50 percent, which has been shown in some reports to decrease the risk of bleeding. This dose reduction strategy has been supported by in vitro data; although there have been no clinical trials supporting that, this approach prevents clinically relevant bleeding [2]. An additional approach includes quantitative assessment of platelet function to guide decision making on the initiation and continued use of ibrutinib [11, 12]. In regards to concurrent antiplatelet therapy, clinicians have been advised to consider stopping aspirin in patients on ibrutinib with low to moderate cardiovascular risk. Dual antiplatelet therapy poses a different scenario given the inhibition of multiple platelet activation and aggregation pathways. In such clinical scenarios, physicians are advised to either temporarily stop ibrutinib therapy or consider alternative therapeutic treatments. To our knowledge, this is the only case of hemorrhagic cardiac tamponade caused by ibrutinib and aspirin therapy. Currently, ibrutinib is front line therapy in many countries. We expect that its increased use will result in more bleeding complications. This case highlights the need for caution when using antiplatelet therapies and ibrutinib together. Further trials are needed to stratify risks and guide continued use of both antiplatelet and anticoagulants in patients on ibrutinib therapy for CLL treatment.

## 4. Conclusion

Life-threatening hemorrhage associated with ibrutinib therapy is thought to be a rare occurrence. The risk of severe hemorrhage is greater with the concurrent use of antiplatelet or anticoagulant therapies. Bleeding complications will likely be seen more frequently with the increased utilization of ibrutinib as it is now front line therapy in many countries. Specific guidelines concerning concurrent use of these agents with ibrutinib need to be established given the increasing use of ibrutinib and the frequent need for antiplatelet or anticoagulant agents in the CLL population. This case highlights why concurrent ibrutinib and antiplatelet therapy should be avoided in most cases, and when combination therapy is warranted, it should be administered with extreme caution, dose adjustment, and close clinical monitoring.

## Figures and Tables

**Table 1:** Laboratory studies on admission and prior to admission.

Laboratory studies	Outside hospital 5/8/2019	Admission 5/13/2019	Reference ranges
White blood count (WBC)	16.9	16.3	4.0–11.0 × 10^9^/L
Hemoglobin	11.6	10.5	13.7–17.0 g/dL
Platelet count	137	204	150–400 × 10^9^/L
Creatinine	1.1	2.43	0.7–1.2 mg/dL
Alanine transaminase (ALT)	9	93	16–61 U/L
Aspartate transaminase (AST)	21	59	15–37 U/L
Alkaline phosphatase	43	123	45–117 U/L
Prothrombin time (PT)	10.5	15	12.3–14.6 sec
Partial thromboplastin time (PTT)	27.7	29	23–36 sec
International normalized ratio (INR)	1.0	1.2	0.8–1.2
